# Formulation Development and *in vitro* Characterization of Proliposomes for Topical Delivery of Aceclofenac

**DOI:** 10.4103/0250-474X.49119

**Published:** 2008

**Authors:** Vandana Gupta, Ashok K. Barupal, Suman Ramteke

**Affiliations:** School of Pharmaceutical Sciences, Rajiv Gandhi Technical University, Airport Bypass Road, Gandhi Nagar, Bhopal-462036, India

**Keywords:** Aceclofenac, liposome, proliposome, sustained release, transdermal delivery

## Abstract

Non-steroidal antiinflammatory drugs are routinely prescribed for the patients with rheumatic disease and such patients are at increased risk of serious gastrointestinal complications, when non-steroidal antiinflammatory drugs administered by oral route. The aim of the present study was to develop and characterized a vesicular drug carrier system (proliposome) for topical delivery of aceclofenac to overcome the problems related with oral route. Aceclofenac proliposome were prepared by the film-deposition on carriers method and characterized for size and surface morphology, drug content in both proliposomes and liposomal system, percent yield, *in vitro* drug release studies and drug permeation studies. The prepared system was also characterized for drug-excipients interaction by Fourier transform infrared spectrophotometer and stability studies. The size and surface morphology were studied using optical microscopy, scanning electron microscopy and transmission electron microscopy. A spherical shape of reconstituted aceclofenac liposome with an average vesicular size of about 500 nm was observed in photomicrographs. The maximum entrapment efficiency of reconstituted liposomes was 80.31% whereas the drug content in proliposomes was found to be more than 90%. *In vitro* release of drug was significantly retarded indicating sustained release of aceclofenac from proliposomes. Stability study was performed at various temperatures indicating that aceclofenac proliposomes are stable at lower temperature.

Aceclofenac is one of the non-steroidal antiinflammatory drugs (NSAIDs) used for the treatment of rheumatoid arthritis and osteoarthritis which reduces levels of prostaglandin E_2_ in the synovial fluid and suppresses its production by blood polymorphonuclear leukocytes or mononuclear cells^1^. The oral administration of aceclofenac has often resulted in side effects, including gastrointestinal ulcer and anemia due to gastrointestinal bleeding. As an alternative route for the drug, transdermal administration can eliminate these side effects, which also offer many advantages, such as increased patient compliance and the possibility for continuous and controlled drug absorption^2^. Clinical evident suggest that topically applied non-steroidal antiinflammatory drugs are safer and at least as efficacious as oral NSAIDs in the treatment of rheumatic diseases^3^.

Drug delivery from liposomes in transdermal formulation has been studied for many purposes but unstable nature and poor skin permeation limits their use for topical delivery^4^–^6^. Furthermore, problems in the sterilization and large-scale production of liposome remain to be solved^7^–^10^. In order to improve the stability of liposomes, the concept of proliposomes was proposed^11^. Proliposomes offer an elegant alternative to conventional liposomal formulations and are defined as dry, free-flowing particles that immediately form a liposomal suspension when come in contact with water. Because of the solid properties, the stability problems of liposome can be resolved without influencing their intrinsic characteristics. Proliposomes are composed of drug, phospholipid and a water soluble porous powder and can be stored sterilized in a dried state^12^,^13^. Moreover, by controlling the size of the porous powder in proliposomes, relatively narrow range of reconstituted liposome size can be obtained^14^.

Proliposome could be prepared by many methods including crystal-film method^15^, film-deposition on carriers method^16^, fluidized-bed method^17^, powder bed grinding method^18^, freezing and drying method^19^ and spray drying method^20^. In the protocol, based on the laboratory conditions film-deposition on the carrier method was chosen to prepare aceclofenac proliposomes.

## MATERIALS AND METHODS

Aceclofenac was purchased from Ipca laboratories Ltd., Mumbai (India) and soyabean lecithin was purchased from Acros organics, New Delhi (India). Mannitol was purchased from Loba Chem. Pvt. Ltd., Mumbai (India). Reagents and organic solvents such as disodium hydrogen phosphate, potassium dihydrogen phosphate, sodium chloride, sodium hydroxide, methanol and chloroform were purchased from CDH Pvt. Ltd., New Delhi (India) and were all reagent grade or better.

### Preparation of aceclofenac- loaded proliposome:

The proliposome was prepared according to the method reported by Xiao *et al*.^21^ the film-deposition on the carrier method with minor modification. There are various process variables which could affect the preparation and properties of the proliposomes. The optimization of aceclofenac proliposomes was done by preparing the 4 different formulations which vary in amount of lecithin concentration (drug to lecithin ratios of 0.1:0.5, 0.1:1.0, 0.1:1.5, and 0.1:2.0). The effect of amount of lecithin was studied on the entrapment efficiency of drug, percent release of drug, drug permeation study and percent yield. During the preparation of a particular system, the other variables were kept constant. The best result was shown by the formulation comprised of drug to lecithin ratio of 0.1:2.0 which was selected as the optimized formulation. The optimized formulation was further evaluated for optical, scanning and transmission electron microscopy, drug-excipients interaction and for stability. The optimized formulation was prepared by taken 5 g of mannitol powder (sieved with 100 mesh) was placed in a 100 ml round bottomed flask which was held at 60-70° temperature and the flask was rotated at 80-90 rpm. The rotating flask was kept into a water bath at 70-80 rpm and mannitol was dried under vacuum for 30 min. After drying, the temperature of the water bath was adjusted to 20-30°. Aceclofenac (100 mg) and lecithin (2 g) were dissolved in mixed organic solvents (chloroform:methanol, 8:2, v/v) and a 0.5 ml aliquot of the organic solution was introduced into the round-bottomed flask at 37°, after complete drying second aliquots (0.5 ml) of the solution was used up. After the last loading, the flask containing proliposomes was connected to the lyophilizer and subsequently aceclofenac loaded mannitol powders (proliposomes) were placed in a desiccator overnight and then sieved with 100 mesh. The collected powder was transferred into a glass bottle and stored at the freeze temperature until characterization.

### Optical, scanning (SEM) and transmission electron microscopy (TEM):

Surface morphology of proliposomes and plain mannitol particles were examined by a scanning electron microscope (SEM, Leo 430, England). After gold coating of proliposome and plain mannitol particles, their surface morphology was viewed and photographed. Formation of liposomes from proliposomes upon hydration was examined by optical microscopy and transmission electron microscopy (TEM). For optical microscopy, a drop of distilled water was added to the proliposome granules on a glass slide without a cover slip, and the process of liposome formation was observed through an optical microscope at 1000 X. Samples for TEM were prepared by adding a drop of distilled water to aceclofenac proliposome granules and shaking the mixture manually for 2 min. A drop of the resultant liposome suspension was placed onto a carbon –coated copper grid, forming a thin liquid film. The films on the grid were negatively stained by adding immediately a drop of 2% (w/w) ammonium molybdate in 2% (w/v) ammonium acetate buffer (pH 6.8), removing the excess staining solution with a filter paper, and followed by a thorough air-drying. The stained films were then viewed on a transmission electron microscope (TEM, FEI-Philips Tecnai 12) and photographed at 10, 000 X.

### Aceclofenac content in proliposome and reconstituted liposome:

Aceclofenac content in proliposomes was assayed by an UV spectrophotometric method. Proliposomes (100 mg) were dissolved in the mixture of PBS (pH 7.4) and methanol (1:9 v/v ratio) by shaking the mixture manually for 2 min. one ml of the resultant solution was taken and diluted with methanol upto 10 ml and then absorbance was recorded at 275 nm using spectrophotometer. The entrapment efficiency (EE %) of aceclofenac in the reconstituted liposomes was determined after hydration of proliposomes with distilled water. 10 ml of distilled water was added to the 100 mg of proliposome granules and the mixture was shaken manually for 2 min. For the separation of unentrapped aceclofenac the liposomal suspension was subjected to centrifugation on a cooling ultracentrifuge at 5000 rpm for 30 min. The clear supernatant was siphoned off to separate the unentrapped drug. 1 ml of supernatant was taken and diluted with methanol upto 10 ml and absorbance was recorded at 275 nm using UV spectrophotometer. The sediment (1 ml) was resuspended in Triton X-100 (0.2% v/v) and diluted with methanol upto 10 ml and then absorbance was recorded at 275 nm. The % entrapment was determined by following formula: percentage entrapment = amount of aceclofenac in sediment/total amount of aceclofenac added × 100. Amount of aceclofenac in supernatant and sediment gave a total amount of drug present in system.

### Yield of proliposomes:

After complete drying the aceclofenac loaded mannitol powders (proliposomes) were collected and weighed accurately. The yield of proliposomes was determined by formula: percentage yield= total weight of proliposomes/total weight of drug+total weight of added materials×100.

### *In vitro* drug release studies:

The release of drug was determined by using the treated cellophane membrane mounted on the one end of open tube, containing 100 mg of proliposome granules. The dialysis tube was suspended in 100 ml beaker, containing 40 ml PBS (pH 7.4). The solution was stirred at 200 rpm with the help of magnetic stirrer at room temperature. Perfect sink conditions were maintained during the drug release testing. The samples were withdrawn at suitable time interval (at 1, 2, 3, 4, 5, 6, and 24 h). The dissolution medium was replaced with same amount of fresh PBS (pH 7.4) solution to maintain the volume 40 ml through out the experiment. The drug content in the withdrawn samples (1 ml) were estimated at 273.5 nm after making the volume upto 5 ml with PBS (pH 7.4) and cumulative % of drug released was calculated and plotted against time (t).

### Drug permeation studies using rat skin:

Drug permeation study was performed after obtaining the approval of the institutional animals ethical committee of Jawaharlal Nehru Cancer Hospital, Bhopal, Madhya Pradesh, India and in accordance with disciplinary principles and guidelines of the committee for the purpose of control and supervision of experiments on animals (CPCSEA).

Abdominal skin of male wistar rats was used in the study. Rats (250-280 g) were anesthesized slightly by ether and hairs removed from the abdominal skin. The rats were sacrified and the abdominal skin of the rat was separated. The skin was stored at −20° until the permeation study, the skin was hydrated in normal saline at 4° and the adipose tissue layer of the skin was removed by rubbing with a cotton swab.

### Permeation of aceclofenac from proliposome under occlusive condition:

Franz diffusion cell (with effective diffusion area 3.14 cm^2^ and 15.5 ml cell volume) was used for the drug permeation studies. Proliposomes (100 mg) are applied onto the surface of skin evenly. The skin was clamped between the donor and the receptor chamber of diffusion cell. The receptor chamber was filled with freshly prepared PBS (pH 7.4) solution to solubilize the drug. The receptor chamber was stirred by a magnetic stirrer rotating at 300 rpm and kept at 37±2°.

The samples (1.5 ml aliquots) were collected at suitable time interval. Samples were analyzed for aceclofenac content by UV/Vis spectrophotometer at 273.5 nm after making proper dilutions. Cumulative corrections were made to obtain the total amount of aceclofenac permeated at each time interval. The cumulative amount of aceclofenac permeated across the skin was determined as a function of time.

### Drug-excipients interaction studies by FTIR:

The drug-excipients interaction studies were done by FTIR. Drug, lecithin, mannitol and aceclofenac proliposomes were dried and kept in desiccator until analysis. The IR spectra of drug, lecithin, mannitol and aceclofenac proliposome were recorded using Fourier transform infrared spectrophotometer (Jasco FT/IR-470 plus, Japan).

### Stability of aceclofenac proliposomes (drug to lecithin ratio 0.1:2.0):

For the determination of the stability of optimized aceclofenac loaded proliposomes (drug to lecithin ratio 0.1:2.0), formulation was stored in air tight sealed, ambered colored glass containers at various temperatures 8°, room temperature (RT) and 40° for a period of 3 mo. The sample was kept in oven at 40° and in refrigerator at 8°. The aceclofenac proliposomes were sampled at regular time intervals of 30, 60 and 90 d and tested for surface morphology and color changes, residual drug content in proliposomes and entrapment efficiency of reconstituted liposomes.

## RESULTS

### Preparation of aceclofenac-loaded proliposome:

The free-flowing granules of aceclofenac-loaded proliposomes were prepared by the film-deposition on the carrier method. Proliposomes were prepared by the penetration of a methanol-chloroform solution of aceclofenac and lecithin into micropoprous mannitol with subsequent vacuum drying. Compared with mannitol, the appearance of the proliposomes was viscous using sodium chloride and sorbitol as carrier. As a result, mannitol was chosen as the carrier and the mixture of methanol and chloroform was chosen as the solvent. Observation under an optical microscope revealed that proliposomes particles are progressively, but rapidly (i.e. in less than 30 s), converted to form a semi-transparent mixture in water.

### Optical, scanning (SEM) and transmission electron microscopy (TEM):

**Fig. 1:**
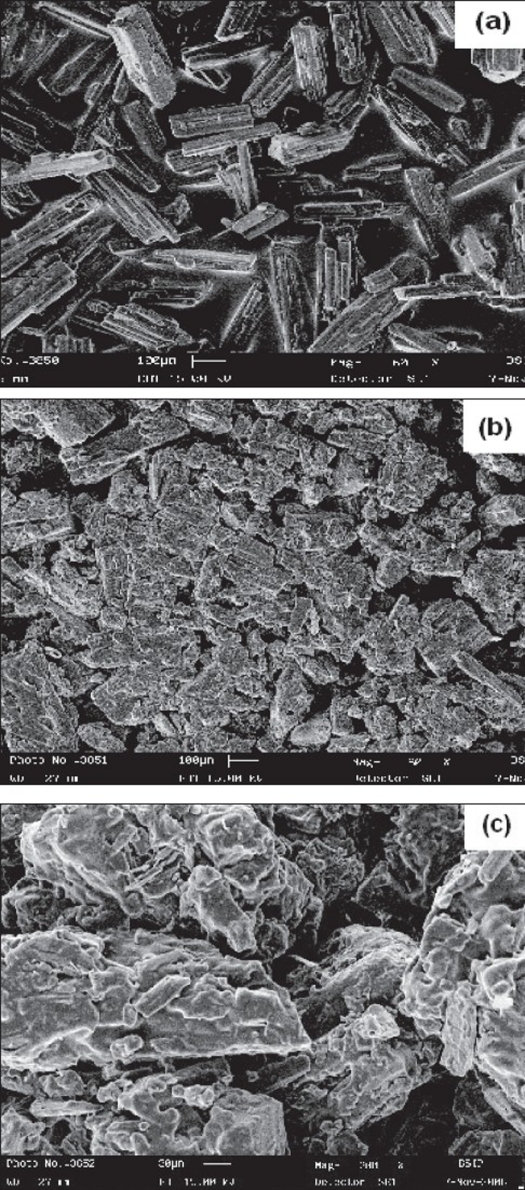
Scanning Electron Microphotographs Scanning electron microphotographs of plain mannitol (a), aceclofenac-loaded proliposomes was recorded at 60 X magnifications (b) and at 200 X magnification to characterize surface properties of the proliposomes (c).

**Fig. 2:**
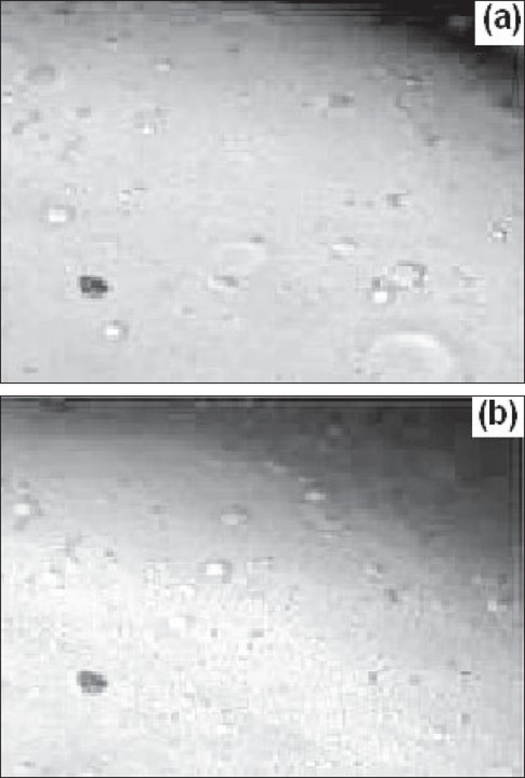
Optical microphotograph of aceclofenac-loaded proliposomes after hydration Optical microphotographs of aceclofenac-loaded proliposomes after hydration at 30 s (a) and at 60 s (b) at the magnification of 1000 X to examine the formation of liposomes.

**Fig. 3:**
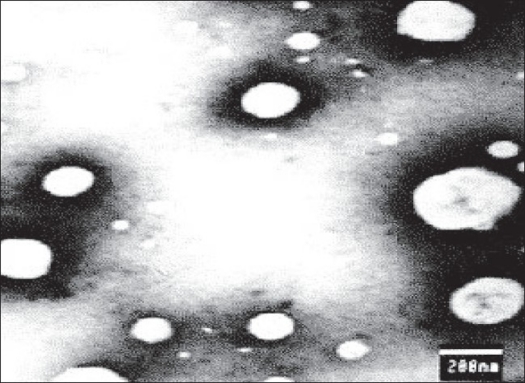
Transmission Electron Microphotographs of aceclofenac-loaded proliposomes after hydration Transmission electron microphotograph of aceclofenac-loaded proliposomes after slightly shaking in distilled water was recorded at 10 000 X magnifications to examine the formation of liposomes.

The surface morphology of proliposome granules and plain mannitol granules were examined by scanning electron microscopy. The surface morphology of proliposome powder was different as compare to plain mannitol powder as shown in SEM (fig. 1). From SEM photographs it is clear that, the surface of mannitol crystals becomes illegible due to deposition of phospholipid on mannitol surface. Observation under an optical microscope revealed that proliposome particles are progressively, but rapidly converted to liposomes within minutes following contact with water (fig. 2). Liposome formation was confirmed again by transmission electron microscopy. A TEM image of the liposomes (reconstituted from the proliposomes) was observed (fig. 3) which confirms that, a suspension of monolayer liposomes with approximate diameter of 200 nm was formed from the proliposomes following contact with water.

### Aceclofenac content in proliposomes and liposomes after hydration:

Aceclofenac content in the proliposomes was determined by UV visible spectrophotometer. The aceclofenac content in the proliposomes were observed in the range of 95.7% to 98.5% at various drug to lecithin ratios. For example, the ratios of 0.1:0.5, 0.1:1, 0.1:1.5 and 0.1:2 resulted in the drug contents of 95.7%, 96.0%, 96.4% and 98.5%, respectively. The % entrapment efficiency of aceclofenac in the reconstituted liposome was determined as described previously. Ten milliliters of purified water was added to 100 mg of proliposomes and the suspension was shaken for two min to form liposome. The entrapment efficiency of aceclofenac in reconstituted liposomes was found to be 50.51±1.94% (mean±SD, n=3), 59.71±2.72%, 64.90±1.81%, 80.31±1.52% as the drug to lecithin ratio in the proliposomes changed from 0.1:0.5, 0.1:1, 0.1:1.5 and 0.1:2, respectively. As the lecithin concentration was increased, it resulted in a corresponding increase in the entrapment efficiency of reconstituted liposomes and aceclofenac content in proliposomes, which indicate that the entrapment of aceclofenac in liposome was found to be dependent mainly on the lipid concentrations.

### Yield of proliposomes:

The % yield of formulations was found to be increase with increase in lecithin concentration. The results of % yield of various formulations were found to be in the range of 89.61±1.54% (mean±SD, n=3), 90.93±1.42%, 91.65±2.79% and 95.82±2.66% as the drug to lecithin ratio in proliposomes was changed.

### *In vitro* release of aceclofenac from proliposomes:

**Fig. 4:**
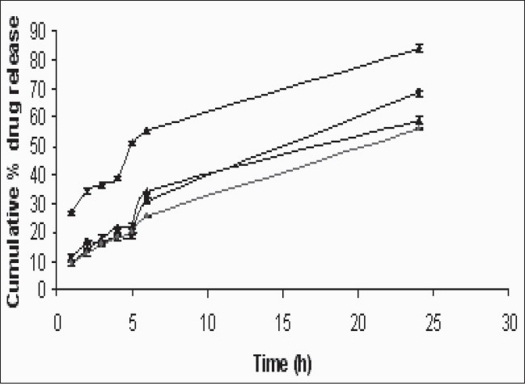
*In vitro* drug release of aceclofenac from proliposomes *In vitro* drug release profiles of aceclofenac from proliposome formulations containing drug/lecithin ratios of 0.1/0.5 (-◆-), 0.1/1.0 (-■-), 0.1/1.5 (-▲-) and 0.1/2.0 (-▪-) in pH 7.4 phosphate buffer saline. Each point represents mean±SD of three different determinations.

The mean cumulative percentage of aceclofenac release from the aceclofenac loaded proliposomes of various formulations in PBS (pH 7.4) are given in fig. 4, as a function of time (Table 1). It was seen that increase in the lecithin concentration further retarded the cumulative release of aceclofenac from proliposomes, indicating sustained release of drug for a longer period of time.

**TABLE 1:** *IN VITRO* CUMULATIVE % DRUG RELEASE PROFILE

Time (h)	Cumulative % drug release (mean±SD, n=3) Formulations			
	
	Drug:lecithin (0.1:0.5)	Drug:lecithin (0.1:1.0)	Drug:lecithin (0.1:1.5)	Drug:lecithin (0.1:2.0)
1	26.82±0.6	11.24±0.7	09.24±0.5	09.04±0.6
2	34.45±0.7	16.17±0.5	14.20±0.6	12.61±1.3
3	36.58±1.2	16.29±0.6	17.89±0.8	15.88±0.8
4	38.96±0.7	18.34±1.4	21.59±0.4	18.54±0.5
5	51.34±0.6	18.54±0.8	22.61±0.7	19.91±1.2
6	55.21±0.9	30.49±0.9	33.80±0.5	25.46±0.6
24	84.14±1.5	68.55±0.8	58.95±1.4	55.92±0.5

SD denotes standard deviation

### Drug permeation study:

**Fig. 5:**
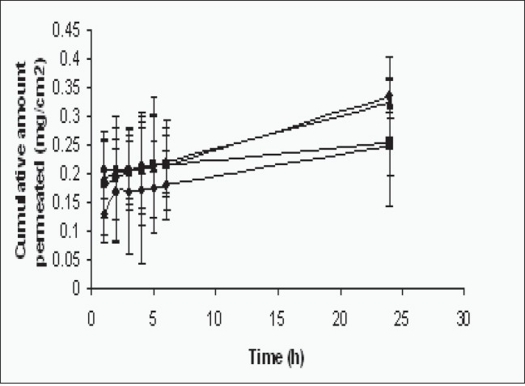
*In vitro* drug permeation profile *In vitro* drug permeation profiles of aceclofenac from proliposome formulation containing drug/lecithin ratios of 0.1/0.5 (-◆-), 0.1/1.0 (-■-), 0.1/1.5 (-▲-) and 0.1/2.0 (-●-) in pH 7.4 phosphate buffer saline. Each point represents mean±SD of three different determinations.

The objective of this study was to examine the feasibility of proliposomes as a transdermal dosage form. The simplest way of topical application may be a direct spread of the proliposomes on the skin followed by occlusive dressing of the dosed site. The permeation of the aceclofenac following application of the proliposomes on the skin under occlusive condition was studied. Fig. 5 and Table 2 show the cumulative amount of aceclofenac permeated from aceclofenac proliposomes to the receptor compartment. PBS (pH 7.4 phosphate buffer saline solution) of the franz diffusion cell, through rat skin.

**TABLE 2:** *IN VITRO* DRUG PERMEATION PROFILE

Time (h)	Cumulative amount permeated (mg/cm^2^, mean±SD, n=3) Formulations			
	
	Drug:lecithin (0.1:0.5)	Drug:lecithin (0.1:1.0)	Drug:lecithin (0.1:1.5)	Drug:lecithin (0.1:2.0)
1	0.130±0.05	0.182±0.09	0.192±0.07	0.205±0.05
2	0.170±0.09	0.192±0.11	0.200±0.08	0.205±0.04
3	0.170±0.11	0.204±0.07	0.206±0.05	0.207±0.06
4	0.172±0.13	0.209±0.08	0.208±0.10	0.211±0.07
5	0.176±0.08	0.214±0.12	0.212±0.09	0.214±0.09
6	0.180±0.06	0.216±0.05	0.220±0.06	0.215±0.08
24	0.248±0.05	0.254±0.11	0.324±0.08	0.336±0.03

SD denotes standard deviation

### Drug-excipients interaction studies by FTIR:

**Fig. 6:**
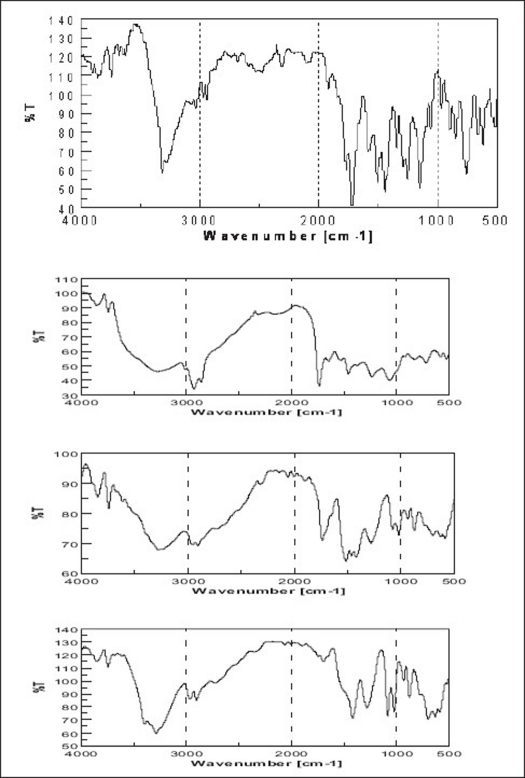
FT-IR Spectra obtained for drug-excipients interaction FT-IR spectra of pure aceclofenac, lecithin (phosphatidylcholine), plain mannitol and aceclofenac loaded proliposomes respectively were recorded to check drug-excipients interaction.

On comparison of IR spectra of proliposomes, plain drug, lecithin and mannitol it was clear that, there was no significant interaction of the encapsulated drug with the lipid component and water soluble solid support (mannitol) in proliposomes (fig. 6).

### Stability of aceclofenac proliposome (drug to lecithin ratio 0.1:2.0):

Stability studies of optimized aceclofenac loaded proliposomes (drug to lecithin ratio 0.1:2.0) was performed at 8°, RT, and at 40° for three mo and analyzed for following parameters: visual appearance, residual drug content, entrapment efficiency of reconstituted liposomes (Table 3). After 3 mo of storage period the aceclofenac proliposomes still appeared free flow and immediately form a liposomal dispersion on contact with water.

**TABLE 3:** STABILITY OF OPTIMIZED ACECLOFENAC PROLIPOSOMES FORMULATION (DRUG:LECITHIN, 0.1:2.0)

Time	Temperature	Residual drug content	Entrapment

(mo)	efficiency	(%, mean±SD, n=3)	(%, mean±SD, n=3)
0	RT	100	100
1	8°	98.33±1.6%	98.93±1.4%
2	8°	96.60±1.8%	97.02±1.6%
3	8°	95.83±1.5%	96.37±1.8%
1	RT	95.82±1.9%	94.27±1.5%
2	RT	94.92±1.6%	90.00±1.8%
3	RT	93.33±2.1%	85.61±2.5%
1	40°	87.50±2.2%	92.59±1.6%
2	40°	85.79±2.5%	77.89±2.4%
3	40°	81.61±2.7%	72.51±1.8%

SD denotes standard deviation

## DISCUSSION

Almost free-flowing granules of aceclofenac-loaded proliposomes could be prepared by the method mentioned above (film deposition on the carrier method). Aceclofenac proliposomes were prepared by the penetration of a chloroform-methanol solution of aceclofenac and lecithin into microporous, water soluble carrier (mannitol) with subsequent drying. In this protocol, lecithin was used as a vesicle forming component, mannitol as a water soluble porous solid support and mixture of methanol-chloroform was used for providing softness to vesicle membrane. Compared with mannitol, the appearance of the proliposomes was viscous using sodium chloride and sorbitol as carrier. As a result, mannitol was selected as the carrier for proliposomes.

In this study, it was found that as the lecithin concentration was increased, it resulted in corresponding increase in the entrapment efficiency of reconstituted liposomes. The phospholipid-rich domains of vesicle might have helped to enhance the percent entrapment of lipophilic drug molecule like aceclofenac in lipid bilayer, which indicate that entrapment of aceclofenac in reconstituted liposome was found to be dependents mainly on the lipid concentration. Increase in lipid concentration in proliposome was also able to control the release of the active for longer period of time, which shows the sustained release behavior of formulations.

In conclusion, a sustained delivery of aceclofenac can be achieved by proliposomal drug delivery system. Phospholipids, being the major component of liposomal system, can easily get integrated with the skin lipids and maintain the desired hydration conditions to improve drug permeation. Fusion of lipid vesicles with skin contributed to the permeation enhancement effect. The lecithin was found to have a significant influence on the lipid matrix of the stratum corneum, suggesting a disruption of the intercellular lipid lamellar structure and act as penetration enhancer. Hence as the lecithin concentration was increased, it would increase the permeation of aceclofenac following application on the skin. The free flowing properties of the proliposomes granules will be beneficial in formulating the proliposomes as a solid dosage form. Proliposomes exhibited superior stability as compared to liposomes and it offers non-invasive delivery of drug into or across the skin. Since better stability of liposomes *in vitro* is observed with proliposomes, the proliposomes would be proper choice of preparation method. Introduction of proliposomes has initiated a new area in vesicular research for topical drug delivery. Different reports show a promising future of proliposomes in making transdermal delivery of various agents.
